# Editorial: Emerging pneumonia and acute lower respiratory infections in children, volume II

**DOI:** 10.3389/fped.2024.1372533

**Published:** 2024-02-01

**Authors:** Hong-Ren Yu, Jong-Hau Hsu

**Affiliations:** ^1^ Department of Pediatrics, Kaohsiung Chang Gung Memorial Hospital, Kaohsiung, Taiwan; ^2^ College of Medicine, Chang Gung University, Taoyuan, Taiwan; ^3^ Department of Pediatrics, Kaohsiung Medical University Hospital, Kaohsiung Medical University, Kaohsiung, Taiwan; ^4^ Department of Pediatrics, School of Medicine, College of Medicine, Kaohsiung Medical University, Kaohsiung, Taiwan

**Keywords:** children, pneumonia, respiratory tract, infection, pediatric

**Editorial on the Research Topic** Emerging pneumonia and acute lower respiratory infections in children, volume II

After the COVID-19 pandemic, there have been some changes in the incidence and influencing factors of childhood pneumonia. Although almost all children with pneumonia associated with COVID-19 have a favorable outcome, there have been long-term consequences similar to other viral pneumonias. Some studies have also indicated an increase in the incidence of childhood pneumonia post-COVID-19, partly attributed to changes in its etiology (1, 2). Additionally, there has been an increase in more severe cases of community-acquired pneumonia in children, possibly due to the reduced exposure to bacterial and viral pathogens and decreased immune system training as a result of non-pharmaceutical interventions implemented during the COVID-19 period, leading to what is known as “immunological debt” (3). Given the changes in the era and environment and the advancements in science, management strategies for childhood pneumonia also need to be based on new evidence and treatment guidelines. Frontiers in Pediatrics has planned “Emerging Pneumonia and Acute Lower Respiratory Infections in Children, Volume II” to highlight the latest developments in this field. This special issue includes 5 articles.

Artificial intelligence (AI) plays a pivotal role in predicting disease prognosis and guiding medical decision-making. By integrating diverse personal data, AI offers more effective disease prevention and treatment decisions, thereby enhancing patient outcomes with precise medical information. Moreover, AI applications in medical imaging can elevate early diagnosis rates, leading to improved treatment efficiency (4).

The nomogram has gained significant application and clinical significance in the field of oncology (5), whereas its application in pediatric respiratory diseases is relatively new. It is essential for clinical practitioners to fully understand the advantages and limitations of using nomograms in clinical practice to achieve better outcomes. Nomograms utilize statistical models to combine multiple predictive factors, providing an intuitive and quantitative prognosis estimate. By inputting specific patient data into the appropriate nomogram model, clinical practitioners can estimate a patient's prognosis to consider treatment strategies.

## A prediction model for the efficacy of continuous positive airway pressure (CPAP) on bronchiolitis

In this article, Shi et al. included 510 children and established a nomogram predictive model consisting of 10 predictor variables, including fever, APTT, white blood cell count, blood potassium concentration, lactate, immunodeficiency, atelectasis, consolidation, congenital airway malformations, and congenital heart disease. This model accurately predicts the likelihood of children with bronchiolitis being able to discontinue CPAP therapy within 48 h. The nomogram predictive model can help clinical staff intuitively understand the multifaceted impact of various factors on the effectiveness of CPAP for bronchiolitis and can be applied in hospitals of different levels.

## A nomogram for predicting severe adenovirus pneumonia in children

In this study, Zhang et al. enrolled 132 patients with non-severe adenovirus pneumonia, of whom 30 progressed to severe adenovirus pneumonia and 102 developed mild adenovirus pneumonia. The researchers utilized respiratory rate, neutrophil percentage, lymphocyte percentage, and LDH as key clinical indicators to construct a nomogram predictive model for predicting the likelihood of severe adenovirus pneumonia. This predictive model aids in early identification of children at risk of progressing to severe adenovirus pneumonia.

Although the aforementioned two predictive models demonstrated excellent discriminative ability as well as high sensitivity and specificity in the retrospective cohort, there are limitations to the nomogram model. First, the construction of the nomogram is based on historical long-term clinical data, and it cannot promptly update the prognosis prediction when patients undergo new treatment regimens or experience new clinical conditions. Second, the nomogram is heavily reliant on accurate clinical data as input, thus it may not accurately predict in situations of insufficient data. Third, the nomogram may consist of multiple variables, leading to complex relationships such as nonlinearity, interaction effects, and multicollinearity. This makes it difficult to intuitively understand the overall structure of the model and the influencing factors, and it is challenging to interpret the prediction process using simple rules. Fourth, it is not appropriate to directly compare the AUC of ROC curves generated using different databases. While the AUC is a discerning indicator of model accuracy, differences in AUC (Area Under the Curve) between ROC curves do not necessarily indicate the clinical significance of each curve. Even with similar variables, AUC may be influenced by different patient groups.

In order to reduce antibiotic overuse and avoid misdiagnosis in the context of pathogen infections in childhood pneumonia, it is recommended to refer to treatment guidelines developed by experts based on local specific patterns of pathogen drug resistance and prevalence (6). Additionally, timely use of bronchoscopy and modern diagnostic techniques can be employed to identify the specific pathogens causing the infection and select appropriate treatment strategies, thus avoiding indiscriminate antibiotic use (7–9). Using the positive association between elevated CRP levels and bacterial pneumonia to appropriately determine the timing for antibiotic utilization. Appropriately utilizing the positive correlation between elevated CRP levels and bacterial pneumonia to determine the timing of antibiotic use can help reduce antibiotic overuse while avoiding ineffective treatment due to inadequate antibiotic use. Certainly, adequate nutrition and comprehensive immunization are also important strategies for reducing the occurrence of childhood pneumonia.

## Metagenomic next-generation sequencing in a diagnosis of Pneumocystis pneumonia in an X-linked immunodeficient child: a case report

Lu et al. reported a 6-month-old male infant with X-linked immunodeficiency presenting with pneumonia and sepsis. When conventional diagnostic methods failed to identify the pathogen, blood metagenomic next-generation sequencing helped identify 133 specific nucleic acid sequences of Pneumocystis pneumonia, indicating the presence of this pathogen infection. This case report highlights the value of metagenomic next-generation sequencing in diagnosing PCP.

## The dilemma of improving rational antibiotic use in pediatric community-acquired pneumonia

In this article, Nguyen et al. conducted a comprehensive review of the disease burden, pathogens, case management, and current available prevention strategies for community-acquired pneumonia in children, with a particular emphasis on the rational use of antibiotics in children pneumonia to stimulate critical assessment of appropriate antibiotic use.

## Recurrent respiratory tract infections in children might be associated with vitamin A status: a case-control study

Zhang et al. investigated the association between vitamin A status in children and recurrent respiratory tract infections (RRTIs), as well as the relationship between dietary vitamin A intake and RRTIs. The results revealed that low serum vitamin A levels were associated with a higher incidence of RRTIs in children, and a low intake of vitamin A-rich foods was also associated with the development of RRTIs. It is unknown whether vitamin A deficiency is directly related to the occurrence of RRTIs, and whether vitamin A supplementation can reduce the occurrence of recurrent respiratory tract infections. Future research should further validate across different ethnic groups and elucidate the molecular mechanisms of vitamin A deficiency leading to RRTIs.
